# The habitat disruption induces immune-suppression and oxidative stress in honey bees

**DOI:** 10.1002/ece3.21

**Published:** 2011-10

**Authors:** Tomomi Morimoto, Yuriko Kojima, Taku Toki, Yayoi Komeda, Mikio Yoshiyama, Kiyoshi Kimura, Keijiro Nirasawa, Tatsuhiko Kadowaki

**Affiliations:** 1 Graduate School of Bioagricultural Sciences, Nagoya UniversityChikusa, Nagoya, Japan; 2 Honey bee Research Unit, Animal Breeding Research Group, Animal and Reproduction Division, National Institute of Livestock and Grassland Science2 Ikenodai, Tsukuba, Ibaraki, Japan; 3 Animal Breeding Research Group, Animal and Reproduction Division, National Institute of Livestock and Grassland ScienceTsukuba, Ibaraki, Japan

**Keywords:** Greenhouse, habitat degradation, honey bee, immune-suppression, oxidative stress, pollination

## Abstract

The honey bee is a major insect used for pollination of many commercial crops worldwide. Although the use of honey bees for pollination can disrupt the habitat, the effects on their physiology have never been determined. Recently, honey bee colonies have often collapsed when introduced in greenhouses for pollination in Japan. Thus, suppressing colony collapses and maintaining the number of worker bees in the colonies is essential for successful long-term pollination in greenhouses and recycling of honey bee colonies. To understand the physiological states of honey bees used for long-term pollination in greenhouses, we characterized their gene expression profiles by microarray. We found that the greenhouse environment changes the gene expression profiles and induces immune-suppression and oxidative stress in honey bees. In fact, the increase of the number of *Nosema* microsporidia and protein carbonyl content was observed in honey bees during pollination in greenhouses. Thus, honey bee colonies are likely to collapse during pollination in greenhouses when heavily infested with pathogens. Degradation of honey bee habitat by changing the outside environment of the colony, during pollination services for example, imposes negative impacts on honey bees. Thus, worldwide use of honey bees for crop pollination in general could be one of reasons for the decline of managed honey bee colonies.

## Introduction

Insect pollination provides a critical ecosystem function that is also necessary for production of a variety of agricultural crops. Approximately 84% of 300 commercial crops are insect pollinated (Richards 1993; Williams 1996). Pollination by insects, primarily honey bees (*Apis mellifera*), remains an essential step in the production of melons, squash, apples, berries, and almonds (Klein et al. 2007). Although other bee species such as bumble bees are also used for crop pollination, the honey bee is the most important commercial pollinator. Thus, the recent decline of managed honey bee colonies in several countries has stirred debate regarding the effects on worldwide crop production (Klein et al. 2007; Aizen et al. 2008, 2009; Allsopp et al. 2008; Aizen and Harder 2009). There are a number of possible causes for the decline, for example, pathogens, parasites, pesticides, and environment (Vanengelsdorp and Meixner 2010; Williams et al. 2010). Although the use of honey bees for pollination could disrupt the habitat of colonies, its effects on honey bees’ physiology have not been determined.

**Figure 1:**
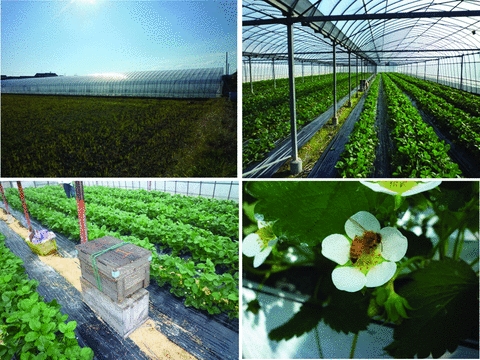
Honey bee colonies installed in greenhouse for pollination. In this study, we characterized the physiological states of honey bees used for long-term pollination in strawberry and eggplant greenhouses.

In Japan, more than 100,000 honey bee colonies are necessary for crop pollination each year. Among these, approximately 80% are used in greenhouses. About 60% of them (approximately 50,000 colonies) are used for pollination of strawberry and the rest are used for pollination of eggplant, melon, and watermelon in greenhouses. The major use of honey bees for pollination in greenhouses is quite specific to Japan (Fig. 1).

A number of reports from honey bee suppliers for crop pollination reveal that honey bee colonies often collapse when they are installed in greenhouses, particularly for pollination of strawberry during winter season (from November to April). If this happens during the pollination period, farmers will have to buy or rent new honey bee colonies, resulting in an increase of production costs. Furthermore, the critical reduction of worker bees makes recycling of colonies, after pollination, difficult for beekeepers. Thus, to suppress colony collapse and maintain a certain number of worker bees in the colony during long-term pollination are major interests of farmers and beekeepers.

To achieve above goal, it is first necessary to understand honey bee physiology during pollination service in greenhouses. The environment inside greenhouses could be deleterious to honey bees’ physiology. Normal habitat of honey bees includes free-flying and visiting various flowers to collect both pollen and nectar. However, flying distance is limited in greenhouses, and honey bees can visit only one type of flower, and most crops such as strawberry and eggplant do not produce nectar. Moreover, worker bees must forage for pollen to nurse their broods in strawberry greenhouses during winter when they are normally in the middle of overwintering. Thus, these can be considered as habitat degradation of honey bees. To understand the effects of these factors on honey bees, we characterized the gene expression profiles of honey bees in the colonies installed in strawberry and eggplant greenhouses during pollination. We have found that the greenhouse environment induces immune-suppression and oxidative stress in honey bees. Consistent with these results, the increase of the load of *Nosema* microsporidia and protein carbonyl content was observed in honey bees during pollination in greenhouses. Physiological disruptions of honey bees used for pollination in greenhouses will be discussed here.

## Methods

### Collection of honey bees from strawberry and eggplant greenhouses

We installed two four-combs colonies (#1 and #2) in two strawberry greenhouses (600 m^2^, one colony per greenhouse) from November 7, 2009 to April 24, 2010. We installed two four-combs colonies (#3 and #4) in an eggplant greenhouse (1500 m^2^) from April 1 to June 9, 2010. We measured the colony weight, photographed all combs in single colonies, and collected worker bees inside hive (approximately 150 individuals) every 2 weeks. The bee samples were immediately frozen with dry ice, and then stored at –80°C. They were first used for preparing total RNA for microarray, and then for quantitative RT-PCR analysis and measurements of protein carbonyl content later. Next year, four four-combs colonies (#5–8) were also installed in four strawberry greenhouses (600 m^2^, one colony per greenhouse) from November 5, 2010 to April 21, 2011 to test and confirm the results obtained with microarray analysis in a previous year. These colonies were processed, and worker bees were collected as described above. For quantitative RT-PCR analysis, examinations of *Nosema* microsporidia, and measurements of protein carbonyl content, we collected worker bees inside hive (approximately 150 individuals) from four seven-combs colonies in an apiary every 2 weeks from November 7, 2009 to April 24, 2010, from April 1 to June 9, 2010, and from November 5, 2010 to April 21, 2011 as controls. The apiary is located at Nagoya University where honey bees can feed loquat and camellia flowers during winter, and plum, magnolia, brassica, and chestnut flowers during spring seasons. All of honey bees were sampled from 1 pm to 3 pm in the day. Strawberry and eggplant greenhouses are located in Yatomi and Nishio, Japan, respectively. All of above colonies used for the experiments were derived from the same beekeeper, suggesting that their genetic backgrounds are similar.

### Microarray and statistical analysis

Fifty worker bees collected from each colony were pooled, and then total RNA was isolated with Trizol reagent (Life Technologies Japan Ltd., Tokyo, Japan), and then purified with High Pure RNA Tissue Kit (Roche Diagnostics Japan, Tokyo, Japan). We verified the quality of isolated RNA using an Agilent 2100 Bioanalyzer (Agilent Technologies Japan Ltd., Tokyo, Japan). Oligonucleotide-based microarrays (UIUC Honey Bee oligo 13K v1) fabricated at the University of Illinois, Keck Center for Comparative and Functional Genomics were used for the experiments. A total of 13,440 probes specific for RNA transcripts were present on the array. RNA (0.2 µg) was used for cDNA synthesis followed by cRNA labeling and amplification with a Low Input Quick Amp Labeling Kit (Agilent Technologies Japan Ltd.). After purification, Cy3-labeled cRNA (2 µg) was fragmented and applied to a prehybridized microarray slide, and then hybridized for 17 hour at 65°C. We sequentially washed arrays with Agilent Gene Expression washing buffer 1 and 2, and then dried them. We scanned them with an Agilent Technologies Microarray Scanner, and then quantified the intensity of each spot using a Feature Extraction 9.5.3.1 (Agilent Technologies Japan Ltd.).

Each RNA sample was hybridized to one array, and thus the results obtained with 16 microarrays were analyzed. Background subtraction and normalization of probe set intensities was performed using the method of Robust Multiarray Analysis (RMA) including a quantile normalization (all arrays are considered to have an equal intensity distribution) described by Irizarry et al. (2003). To identify differentially expressed genes, gene expression intensity was compared using a two-way analysis of variance (ANOVA) test with a significance threshold *P* < 0.01 and a Bayes smoothing approach developed for a low number of replicates (Smyth 2004). To correct for the effect of multiple testing, we estimated a False Discovery Rate (FDR), set at 0.05, from *P*-values derived from the ANOVA test statistics. We performed all statistical analysis with the Partek Genomic Suite 6.5 Software (Partek Inc., MO, USA). We submitted raw and normalized data from microarray experiments to the Gene Expression Omnibus database (http://www.ncbi.nlm.nih.gov/geo). The platform ID is GPL13528. The accession ID is GSE29252.

### Gene ontology (GO) analysis

Functional insights into differentially expressed genes (all > two fold up/downregulated genes [*P* < 0.01]) were obtained by conducting a GO term enrichment analysis. We performed this analysis using the FlyBase identification number representing the best BLAST hit for each honey bee gene (Honey bee Genome Sequencing Consortium 2006). Enrichment in GO terms of biological processes on level 5 (GOTERM_BP5 chart) was determined by using GOToolBox (Martin et al. 2004) with a hypergeometric test followed by FDR correction for multiple testing (GO categories at *P* < 0.05 are shown). For each experiment, the reference gene set corresponded to the total number of genes analyzed on the microarray.

### Quantitative RT-PCR analysis

Total RNA (1 µg) purified for microarray analysis and isolated from 50 pooled honey bees of each colony installed in strawberry greenhouse (#5–8 as above, four colonies in total), and each control colony in an apiary (four colonies in total) was used for reverse transcription with random hexamer primer and ReverTra Ace reverse transcriptase (TOYOBO, Osaka, Japan). Primer pairs (Table S11) were designed to produce 120- to 180-bp products using Primer-BLAST (http://www.ncbi.nlm.nih.gov/tools/primer-blast/index.cgi?LINK_LOC=BlastHome). We quantified PCR reactions using SYBR Green I (KAPABIOSYSTEMS) and the enzyme activation was first carried out at 95°C for 20 sec. The following reaction with 40 cycles of a thermal profile consisting of 95°C for 3 sec and 60°C for 30 sec was performed with StepOnePlus (Applied Biosystems), and expression was normalized against the geometric mean CT (cycle threshold) value for two honey bee housekeeping genes (Beta-actin and RP49).

### PCR detection of *Nosema* microsporidia and counting the spores in honey bees

Total genomic DNA was isolated from 20 honey bees of a single colony using DNAzol reagent (Life Technologies Japan Ltd.), and dissolved in 100 µl of 8 mM NaOH followed by neutralization by adding 1 µL of 1 M HEPES. Total DNA (0.1 µg) was used for PCR with KOD FX DNA polymerase (TOYOBO) and the following primer sets: 5′-CCATTGCCGGATAAGAGAGT-3′ and 5′-CCACCAAAAACTCCCAAGAG-3′ for *Nosema apis*, and 5′-CGGATAAAAGAGTCCGTTACC-3′ and 5′-TGAGCAGGGTTCTAGGGAT-3′ for *N. ceranae* (Chen et al. 2009). As a control, a honey bee genomic DNA fragment encoding a part of *AmHsTRPA* (Kohno et al. 2010) was PCR amplified with the following primers: 5′-CACGACATTCAAGGTTTAAGAAATCACG-3′ and 5′-TCA GTTATTCTTTTCCTTTGCCAGATTT-3′. The thermal cycling conditions were as follows: one cycle of initial denaturation at 94°C for 2 min, 35 cycles of denaturation at 98°C for 10 sec, annealing at 55°C for 30 sec, and extension at 68°C for 30 sec. The PCR product was analyzed by 2% agarose gel electrophoresis. A negative control lacking template DNA was performed for each PCR reaction. Positive DNA controls were not included to eliminate the possibility of contamination. Positive identification was confirmed by sequencing the PCR products.

Abdomens of 10 honey bees from each colony (#5–8 as above) were homogenized with 10 mL PBS (phosphate buffered saline), and then the microsporidian spores were counted by light microscopy (magnification, ×400) with hemocytometer. The spore counting was repeated twice as above with additional 10 honey bees derived from the same colony (20 honey bees in total). The rather moderate sampling size does not allow the detection of the less infected bee in the colony but does allow the detection of an infection level above ∼15% at the 5% significance level (Fries et al. 1984), which can be considered biologically relevant (Higes et al. 2008).

### Measurement of protein carbonyl

Twenty honey bees from each colony installed in strawberry (#1 and #2 as above) and eggplant (#3 and #4 as above) greenhouses as well as each control colony in an apiary were homogenized in 10 mL of 5 mM phosphate buffer (pH 7.5) containing a protease inhibitor cocktail (Roche Diagnostics Japan). After centrifugation, we treated 350 µl of the supernatant with an equal volume of 2 M HCl (control) or 0.2% (w/v) 2, 4-dinitrophenylhydrazine (DNPH) in 2 M HCl. After incubation at room temperature for 1 hour, the samples were precipitated with an equal volume of 20% trichloroacetic acid (TCA). We washed the precipitates once with 10% TCA, then thrice with ethanol/ethyl acetate (1:1, vol/vol). The samples were then dissolved in 1 mL of 6 M guanidine/20 mM phosphate buffer (pH 6.5) and debris was removed by centrifugation. We measured the absorbance at 366 nm and the difference between DNPH- and HCl-treated samples was converted into nanomol of carbonbonyl groups per milligram of protein using 22.0 mM^−1^ cm^−1^ as extinction coefficient (Levine et al. 1990). We measured the protein concentration in the HCl-treated samples by the BCA (bicinchoninic acid) method. We repeated these experiments twice with 20 honey bees derived from the same colony.

## Results

### Changes in colony weights during pollination in strawberry and eggplant greenhouses

**Figure 2:**
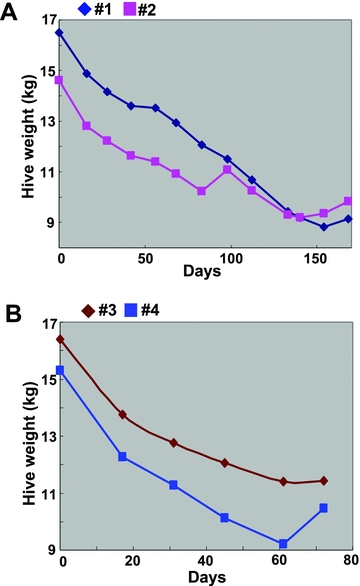
Changes in colony weight during pollination of strawberry and eggplant in greenhouses. Weights of colonies installed in strawberry (A; colony #1 and #2) and eggplant (B; colony #3 and #4) greenhouses were measured every 2 weeks. Colony weights steadily decreased during pollination in the greenhouses. The weight increase of colony #2 in the strawberry greenhouse at day 98 was due to a supply of honey bee diet.

Two colonies (#1 and #2) were installed for pollination service in two strawberry greenhouses (one colony/a greenhouse) from November 7, 2009 to April 24, 2010. Two colonies (#3 and #4) were placed for pollination in an eggplant greenhouse (two colonies/a greenhouse) from April 1 to June 9, 2010. We characterized these two different honey bee samples to examine whether the greenhouse environment induced the same effects on honey bee physiology despite of the differences of size, inside temperature, crop species, and season in a year between strawberry and eggplant greenhouses. As shown in Fig. 2A and 2B, the colony weights were steadily reduced during pollination. At 168 days after installation in strawberry greenhouses, colonies lost 33–45% of their initial weight. The weight increase of colony #2 in the strawberry greenhouse at day 98 was due to supplying this colony with a diet for honey bees. At 70 days after installation in the eggplant greenhouse, colonies lost 30–32% of their initial weight. Although it was difficult to count the total number of worker bees in colonies with four combs, worker bee density became less during pollination. Appendices A1 and A2 show the four combs of the colony placed in the strawberry greenhouse on November 7, 2009 and February 27, 2010, respectively. We have also begun to investigate four new colonies placed in strawberry greenhouses from November 5, 2010 to April 21, 2011. The reductions in colony weights and worker bees are similar to those observed in the previous year (Appendix A3).

### Changes in gene expression profiles of honey bees during pollination in strawberry and eggplant greenhouses

**Figure 3:**
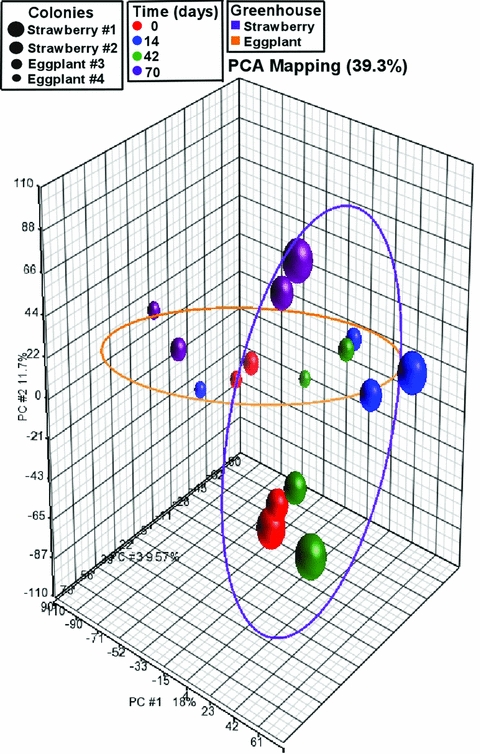
Principle component analysis of microarray results. The red, blue, green, and purple oval dots with different sizes represent linear combinations of the expression data, including relative expression value and variance, of the 13,440 genes in honey bees from the colonies (Strawberry #1 and #2; Eggplant #3 and #4) at 0 (red), 14 (blue), 42 (green), or 70 (purple) days after the installation in the greenhouses. Each colony is indicated by the different oval dot size. Data from strawberry and eggplant greenhouses are encompassed with purple and yellow lines, respectively. The principle component analysis examined three components of genes in different samples for those with similar or different expression profiles. The first component, shown in the x-axis, includes genes with a high degree of variance. The second component, displayed in the y-axis, encompasses genes that had a median range of variance. The third component, represented by z-axis, contains those with a minor variance.

**Figure 4:**
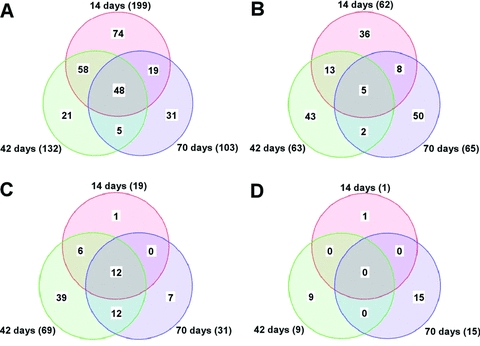
Venn analysis of the up- or downregulated honey bee genes during pollination in strawberry and eggplant greenhouses. The down- and upregulated honey bee genes at 14, 42, and 70 days after the installation of colonies in the strawberry greenhouses are analyzed by Venn diagrams in (A) and (B), respectively. The total numbers of such genes are in parentheses. The numbers of genes in specific regions of overlap are also shown. Similarly, the down- and upregulated honey bee genes at 14, 42, and 70 days after the installation of colonies in the eggplant greenhouses are analyzed by Venn diagrams in (C) and (D), respectively. More genes are down- and upregulated in honey bees of colonies installed in strawberry than eggplant greenhouse. Downregulated genes are greater (in number) than upregulated genes in honey bees of colonies installed in strawberry and eggplant greenhouses.

We analyzed gene expression profiles of honey bees in the colonies at 0, 14, 42, and 70 days after installation in strawberry (#1 and #2) and eggplant (#3 and #4) greenhouses by microarray. Since it was not possible to collect the age-matched worker bees, we analyzed 50 randomly collected honey bees of unknown age. Since each honey bee array contained 13,440 probe sets, we generated 215,040 datapoints from the 16 arrays. We performed Principle Component Analysis (PCA) to examine the correlations among the data produced from different arrays. The results of the first three principal components, which included the variance of 39.3% of the expression datapoints of each sample, are shown (Fig. 3). Each dot represents the overall expression pattern of each honey bee sample. The sample colonies and sampling times are indicated by using different dot sizes and colors, respectively. The PCA showed that duplicated samples (#1 and #2; #3 and #4) at the different time points were closely clustered together except #3 and #4 at 14 days, indicating that the global expression profiles of duplicated honey bee samples are similar irrespective of the possible age differences. The gene expression profiles of honey bees change significantly in a time-dependent manner, and the changes are more dramatic with honey bees in strawberry than in eggplant greenhouses (Figs. 3 and 4). Nevertheless, the more significant source of gene expression variations is the type of greenhouse (strawberry or eggplant) (Fig. 3). We identify and list all differentially expressed genes with a *P* < 0.01 and a fold change (FC) > 2 during pollination period (at 14, 42, and 70 days) relative to prior to colony installation (0 day) in strawberry and eggplant greenhouses (Tables S4 and S5). Venn diagrams show the number of honey bee genes down- or upregulated during the pollination period in strawberry and eggplant greenhouses (Fig. 4). As expected from PCA data (Fig. 3), more genes are differentially expressed with honey bees in the strawberry (624 genes) than in the eggplant greenhouses (144 genes) (*X*^2^= 309.27; *P* < 0.00001). A total of 199, 132, and 103 genes are downregulated at 14, 42, and 70 days after installation in strawberry greenhouses, respectively (Fig. 4A). Among them, 130 genes are common between two or three groups. Meanwhile, 62, 63, and 65 genes are upregulated at 14, 42, and 70 days after installation into the strawberry greenhouses, respectively (Fig. 4B). Thus, the numbers of upregulated genes are less than those of downregulated genes. Furthermore, only 28 genes occur between two or three groups. The ratio of shared genes is higher among the downregulated genes than the upregulated genes (*X*^2^= 44.72; *P* < 0.00001). A total of 19, 69, and 31 genes are downregulated at 14, 42, and 70 days after installation in the eggplant greenhouse, respectively (Fig. 4C). Among them, 30 genes occur between two or three groups. Only 1, 9, and 15 genes are upregulated at 14, 42, and 70 days after installation in the eggplant greenhouse, respectively (Fig. 4D). None of these genes are shared, and thus the ratio of shared genes is higher among the downregulated genes than the upregulated genes (*X*^2^= 13.80; *P* < 0.0002) similar to honey bees placed in the strawberry greenhouses. The common honey bee genes downregulated between the different time points during pollination in the strawberry and eggplant greenhouses include genes associated with antioxidant functions, protein translational activity, and immune system (see below).

**Figure 5:**
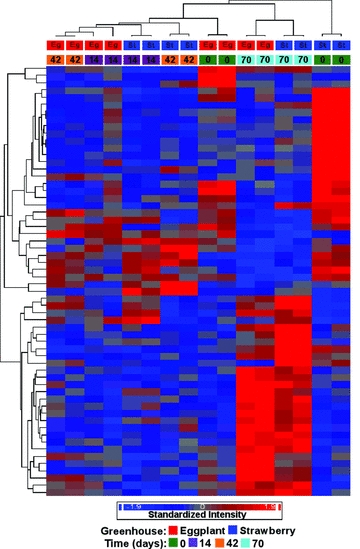
Hierarchical clustering of differentially expressed honey genes during pollination of strawberry and eggplant greenhouses. We performed ANOVA to identify 59 genes that differentially expressed during pollination of strawberry and eggplant greenhouses (FDR < 0.05; FC > 2). The expression profiles in honey bees from the colonies at 0 (green), 14 (purple), 42 (yellow), or 70 (light blue) days after the installation in strawberry (blue) and eggplant (red) greenhouses are clustered. The rows of the heat map represent genes and the columns represent groups for two independent replicate experiments. The colors indicate the fold change relative to the average expression of all genes on the array. Red indicates increased expression, and blue indicates decreased expression. Gray color indicates no change in expression levels. The gene expression profiles of honey bees used for pollination of strawberry and eggplant for 70 days in the greenhouses cluster.

Hierarchical clustering analysis using expression values for 59 differentially expressed genes with an FDR < 0.05 and a FC > 2 shows that the data from strawberry and eggplant greenhouses cluster together at 70 days (Fig. 5). It demonstrates that long-term pollination in greenhouses induces the same changes in expression profiles of particular gene sets irrespective of the crop, greenhouse, and seasonal differences.

### GO analysis of differentially expressed honey bee genes during pollination in strawberry and eggplant greenhouses

The results of GO analysis of honey bee genes differentially expressed during pollination in the strawberry and eggplant greenhouses are in Appendices A6 and A7, respectively. GO terms associated with antioxidant systems such as oxidoreductase activity, response to reactive oxygen species (ROS), glutathione transferase activity, hydrogen peroxide metabolic process, hydrogen peroxide catabolic process, cellular response to oxidative stress, and thioredoxin peroxidase activity are enriched (Appendix A6). This result suggests that antioxidant functions decrease in honey bees used for pollination of strawberry in greenhouses. Furthermore, GO terms such as translation elongation factor activity, translation, and translation factor activity and nucleic acid binding are enriched, suggesting that protein translation activity is also downregulated. In honey bees placed in the eggplant greenhouse, GO terms related to immune system (for example, innate immune response) and vesicle-mediated transport are enriched (Appendix A7), suggesting that immune functions as well as vesicular transport activity are reduced. The results of GO analysis of down- and upregulated honey bee genes during pollination in the strawberry greenhouses are also shown in Tables S8 and S9, respectively. GO terms involved with actin cytoskeleton organization and reproductive process appear to be enriched in the upregulated honey bee genes during pollination in the strawberry greenhouses. Similarly, the result of GO analysis of downregulated honey bee genes during pollination in the eggplant greenhouse is in Table S10. The results obtained with the honey bee genes downregulated during pollination in the strawberry and eggplant greenhouses are basically the same as above. Since the number of upregulated honey bee genes during pollination in the eggplant greenhouse were small (Fig. 4D); they were not subjected to GO analysis.

### Downregulation of immune and antioxidant system genes, cytochrome P450 family genes, and genes associated with proteasome-dependent protein degradation in honey bees used for pollination of strawberry and eggplant in greenhouses

**Figure 6:**
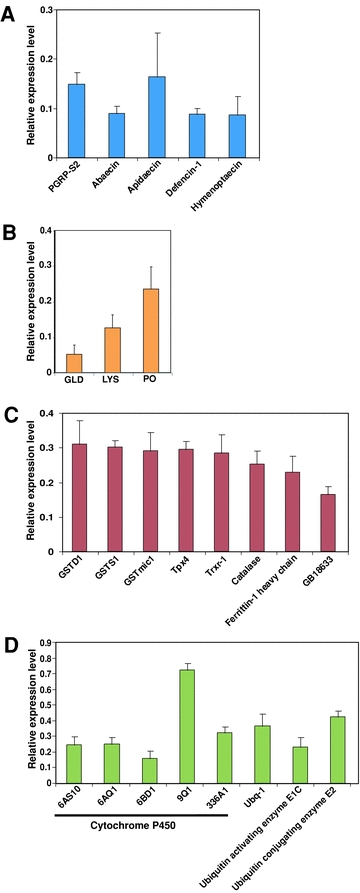
Quantitative RT-PCR analysis of immune and antioxidant system genes, cytochrome P450 family genes, and genes associated with proteasome-dependent protein degradation in honey bees used for pollination of strawberry and eggplant in greenhouses. Relative expression levels of mRNAs for immune (A; PGRP-S2, Abaecin, Apidaecin, Defencin-1, and Hymenoptaecin, B; GLD, LYS, and PO), antioxidant system genes (C; GSTD1, GSTS1, GSTmic1, Tpx4, Trxr-1, Catalase, Ferrittin-1 heavy chain, and GB18633), cytochrome P450 family genes, and genes associated with proteasome-dependent protein degradation (D; Cytochrome P450 6AS10, 6AQ1, 6BD1, 9Q1, 336A1, Ubq-1, Ubiquitin-activating enzyme E1C, and Ubiquitin-conjugating enzyme E2) in honey bees at 42 days after the installation of colonies in strawberry and eggplant greenhouses are measured by quantitative RT-PCR. The mRNA levels in honey bees prior to colony installation in the greenhouses represent 1. All of the values shown are mean ± SEM (A, C, D; *n*= 8, B; *n*= 6). They are significantly different from each control (*t*-test; *P* < 0.002). GLD, LYS, and PO mRNA levels were derived from data obtained with honey bees used for pollination of strawberry in greenhouses.

GO analysis of the above demonstrates that immune and antioxidant system genes of honey bees are significantly enriched in downregulated genes during pollination in greenhouses. We attempted to confirm the downregulation of several immune and antioxidant system genes in honey bees at 42 days after installation in the strawberry and eggplant greenhouses (with colonies #1–8) by quantitative RT-PCR. The decrease of mRNAs for PGRP-S2, a peptidoglycan recognition protein detecting bacteria (Evans et al. 2006), and antimicrobial peptides, Abaecin, Apidaecin, Defencin-1, and Hymenoptaecin (Evans et al. 2006), is confirmed by quantitative RT-PCR (Fig. 6A). Thus, humoral immune functions are downregulated. The suppression of cellular immune functions is also demonstrated by the decrease of mRNAs for glucose dehydrogenase (GLD) in hemocytes which is hypothesized to be required for killing pathogens (Lovallo and Cox–Foster 1999), lysozyme (LYS) hydrolyzing the peptidoglycan of bacterial cell wall (Gillespie et al. 1997), and phenol oxidase (PO) catalyzing melanization (Decker and Jaenicke 2004) in honey bees used for strawberry pollination in greenhouses (Fig. 6B). However, this was not observed with honey bees of colonies installed in the eggplant greenhouse (data not shown). Similarly, mRNAs for antioxidant system proteins (Claudianos et al. 2006; Corona and Robinson 2006), Glutathione S-transferase 1 (GstD1), Glutathione S-transferase S1 (GstS1), Microsomal glutathione S-transferase (GSTmic1), Peroxiredoxin 2540 (Tpx4), Thioredoxin reductase 1 (Trxr-1), Catalase, and Ferritin 1 heavy chain are also decreased (Fig. 6C). GSTs are a family of enzymes that catalyze the conjugation of reduced glutathione to a variety of electrophilic substances (Hayes et al. 2005). Tpx is a type of peroxidase that reduces H_2_O_2_ using electrons provided by Trxr (Chae et al. 1994). Trxr is an essential enzyme that produces Trx (SH)_2_ and GSH, thiol-based reductants, and powerful intracellular antioxidants (Holmgren 1989), from thioredoxin (TrxS_2_) and GSH disulphide (GSSG) (Nordberg and Arner 2001). Catalase prevents free hydroxyl radical formation by breaking down H_2_O_2_ into oxygen and water. Ferritin 1 heavy chain binds and stores iron in a nontoxic form to prevent the formation of free radicals from ROS via the Fenton reaction (Rival et al. 2009). Furthermore, mRNAs encoding several cytochrome P450 proteins (6AS10, 6AQ1, 6BD1, 9Q1, and 336A1), factors associated with proteasome-dependent protein degradation (Ubiquitin-1, Ubiquitin-activating enzyme E1C, and Ubiquitin-conjugating enzyme E2), and GB18633 are downregulated (Fig. 6D). GB18633 appears to be an ortholog of senescence marker protein 30 (SMP30)/regucalcin, a marker protein of aging that plays an important role in intracellular Ca^2+^ homeostasis, ascorbic acid biosynthesis, and oxidative stress in mammals (Maruyama et al. 2010). We did not observe significant downregulation of mRNAs for the above proteins when we compared honey bee samples from four control colonies in an apiary on November 7 and December 19, 2009, as well as on April 1 and May 12, 2010 (data not shown).

### Increase of the load of *Nosema* microsporidia infecting honey bees during pollination in strawberry greenhouses

Immune-suppression during pollination predicts that pathogen loads in the honey bees may increase if they were previously infested prior to installation in greenhouses. To test this possibility, we examined *Nosema* microsporidia that is prevalent (64%) in Japanese *A. mellifera* colonies (Kojima et al., unpubl. manuscript). In fact, it was found that four colonies (#5–8) installed in strawberry greenhouses from November 5, 2010 to April 21, 2011 were positive for *N. ceranae* by genomic PCR detection (data not shown). As shown in Table 1, the number of *N. ceranae* spores/bee with honey bees in all colonies within 42 days after the installation is low (< 10^4^; usually uncountable with our method); however, it increases > 10^6^ with honey bees in all colonies after 84 days except colony #7 at 84 days. Consistent with the immune-suppression, the number of *N. ceranae* infecting honey bees increases during long-term pollination in greenhouses. We did not detect increase in the number of *Nosema* microsporidia in honey bees of four control colonies in an apiary on November 5, 2010 to April 21, 2011. The number of *N. ceranae* spores/bee with honey bees in all control colonies was less than 10^4^ at all time points.

**Table 1:** The number of *N. ceranae* spores/bee with honey bees in colonies installed in strawberry greenhouses (November 5, 2010–April 21, 2011)

	Colony			

Days	#5	#6	#7	#8
0	< 10^4^	< 10^4^	< 10^4^	< 10^4^
14	< 10^4^	< 10^4^	< 10^4^	< 10^4^
28	< 10^4^	< 10^4^	< 10^4^	< 10^4^
42	< 10^4^	< 10^4^	< 10^4^	< 10^4^
56	< 10^4^	2.8 ± 0.2 × 10^6^	< 10^4^	1.6 ± 0.1 × 10^5^
84	7.0 ± 0.4 × 10^6^	6.1 ± 0.3 × 10^6^	< 10^4^	4.9 ± 0.2 × 10^6^
112	2.8 ± 0.3 × 10^6^	8.7 ± 0.5 × 10^6^	1.3 ± 0.08 × 10^7^	9.3 ± 0.8 × 10^6^
140	1.3 ± 0.04 × 10^7^	6.8 ± 0.7 × 10^6^	1.1± 0.07 × 10^7^	3.7 ± 0.04 × 10^6^
168	1.2 ± 0.08 × 10^7^	1.0 ± 0.06 × 10^7^	6.7 ± 0.2 × 10^6^	1.1 ± 0.05 × 10^7^

The number of *N. ceranae* spores was counted in homogenates prepared from abdomens of 10 honey bees. Counting was repeated twice with homogenates prepared from abdomens of additional 10 honey bees. If there were no visible spores, we estimated the number of *N. ceranae* spores/bee should be < 10^4^.

All of the values shown are mean ± SEM (*n*= 6).

### Accumulation of protein carbonyl in honey bees during pollination in greenhouses

**Figure 7:**
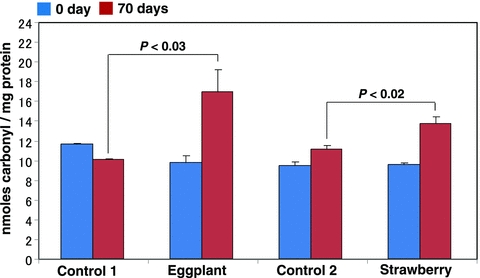
Protein carbonyl contents in honey bees from control colonies and colonies installed in eggplant and strawberry greenhouses. The total amounts of protein carbonyl were measured and compared in honey bees from colonies prior to (0 day) and at 70 days after the installation in eggplant and strawberry greenhouses. They were also measured and compared in honey bees from four control colonies in an apiary on April 1 (0 day) and June 9 (70 days), 2010 (Control 1) as well as on November 7, 2009 (0 day) and January 16, 2010 (70 days) (Control 2). All of the values shown are mean ± SEM (Control 1 and Control 2; *n*= 8, Eggplant and Strawberry; *n*= 4). Comparisons between Control 1 and Eggplant (*t*-test; *P* < 0.03) as well as Control 2 and Strawberry (*t*-test; *P* < 0.02) at 70 days indicate that more accumulation of protein carbonyl occurred in honey bees during pollination in greenhouses.

The downregulation of antioxidant system genes demonstrates the lower detoxification potentials for oxidants in honey bees used for pollination in greenhouses. ROS damage cellular components, such as proteins and DNA (Imlay 2003). In addition, cells accumulate cytoplasmic protein carbonyl (carbonylation of arginine and lysine residues of proteins by Fe^2+^ and ROS) under oxidative stress (Stadtman and Oliver 1991). We thus measured the amount of total protein carbonyl in honey bees placed in greenhouses for 70 days (Fig. 7). The amount of total protein carbonyl increased in honey bees placed in both eggplant (*t*-test; *P* < 0.02) and strawberry (*t*-test; *P* < 0.001) greenhouses relative to that in honey bees prior to installation (0 day). The amount of total protein carbonyl slightly deceased when we compared honey bee samples from four control colonies at an apiary on April 1 (0 day) and June 9 (70 days), 2010 (Control 1; *t*-test; *P* < 0.005). The amount of total protein carbonyl slightly increased when we compared honey bee samples from four control colonies at an apiary on November 7, 2009 (0 day) and January 16, 2010 (70 days) (Control 2; *t*-test; *P* < 0.02). Comparisons between Control 1 and Eggplant (*t*-test; *P* < 0.03) as well as Control 2 and Strawberry (*t*-test; *P* < 0.02) at 70 days indicate that more accumulation of protein carbonyl occurred in honey bees during pollination in greenhouses.

## Discussion

Many honey bee colonies are used for pollination (approximately 6 months) of strawberry in greenhouses during winter in Japan. They are also used for pollination (approximately 2 months) of eggplant in greenhouse during spring. Both strawberry and eggplant flowers produce little nectar, and this may have resulted in the weight loss of colonies installed in greenhouses (Fig. 2; Appendix A3). Unlike colonies in an apiary during winter, the colonies in strawberry greenhouses contain broods that activate foraging of worker bees for strawberry pollen. These broods are necessary to replace dead adult worker bees; however, their number is relatively small, and thus the total worker bee population decreases during pollination (Appendices A1 and A2).

The gene expression profiles of honey bees in colonies installed in greenhouses significantly changed in a time and greenhouse-type (strawberry or eggplant) dependent manner (Figs. 3 and 5). There are a number of different parameters between the strawberry and eggplant greenhouses, for example, size, inside temperature, and crop species. We are not able to determine which factor is most significant at this point. Moreover, the gene expression profiles are quite different between honey bees in the colonies prior to installation in the strawberry and eggplant greenhouses (Figs. 3 and 5). This may represent the seasonal differences in gene expression since honey bees in early winter and mid spring are used for pollination of strawberry and eggplant, respectively.

As shown in Figure 4, the fraction of honey bee genes that exhibit significant expression changes during pollination is small relative to other microarray studies (e.g., Naeger et al. 2011). The gene expression profiles are quite different between honey bees used for pollination of strawberry and eggplant in the greenhouses; however, they become similar at 70 days after the installation (Fig. 5). The greenhouse environments appear to give the specific effects (mainly by downregulating the particular genes) on honey bee physiology. In fact, GO analysis and quantitative RT-PCR analysis demonstrate that the immune and antioxidant system genes, cytochrome P450 family genes, and genes associated with proteasome-dependent protein degradation are downregulated in honey bees used for pollination in the greenhouses (Fig. 6). Decrease of mRNAs for both humoral and cellular immune system genes suggests that signaling pathways associated with immunity (Evans et al. 2006) are downregulated. Honey bees are likely to become more susceptible to pathogen infection during pollination in greenhouses. Immune-suppression of honey bees is also reported with *Varroa* mite parasitism (Yang and Cox–Foster 2005) and *Nosema* microsporidia infection (Antúnez et al. 2009). Therefore, various factors affect honey bee immunity. Nevertheless, their mechanisms remain to be determined.

Genes associated with the antioxidant system are also downregulated in honey bees used for pollination in the greenhouses (Fig. 6C). The mRNA level for honey bee ortholog of SMP30/regucalcin also decreased, suggesting that long-term pollination in greenhouse reduces the potential to detoxify ROS and thus imposes honey bees oxidative stress. Consistent with this prediction, more protein carbonyl accumulates in honey bees during pollination in greenhouses (Fig. 7). These are the representative markers for senescence of various animal species (Stadtman and Oliver 1991; Maruyama et al. 2010), and thus honey bees used for pollination in greenhouses are likely to undergo accelerated senescence compared to those in colonies in an apiary. The mechanisms of honey bee senescence is well studied since queens live approximately 10 times longer than worker bees, and worker bees show the task-dependent aging plasticity (Münch and Amdam 2010). The expression of genes associated with antioxidant system was examined in both queen and worker bees during normal aging process, and it generally decreased with age in queens, but not in workers (Corona et al. 2005). Queen bee longevity may have evolved by mechanisms other than antioxidant system. Later, a yolk protein, vitellogenin (Vg) was shown to act as an antioxidant to promote longevity in both queen and worker bees (Seehuus et al. 2006b; Corona et al. 2007). It was also reported that the protein carbonyl level in the honey bee brain was high in forager (with low Vg titer) than nurse or winter bees (with high Vg titer) irrespective of chronological age (Seehuus et al. 2006a). We thus measured Vg mRNA levels in honey bee workers used for pollination in the greenhouses by quantitative RT-PCR, and found that they did not change during pollination period (data not shown). Thus, Vg may not be important in senescence of worker bees used for pollination in greenhouses. Downregulation of genes associated with proteasome-dependent protein degradation (Fig. 6D) suggests that degradation of damaged proteins by ROS is impaired, leading to further accumulation (Grillari et al. 2006). Senescence may accelerate due to the accumulation of oxidative damage when honey bees are used for pollination in greenhouses.

Immune-suppression and accelerated senescence by oxidative stress might explain why many colonies collapse rapidly when introduced in greenhouses for pollination. Viruses (BQCV, DWV, IAPV, and SBV) and *N. ceranae* are prevalent in *A. mellifera* colonies in Japan (Kojima et al., unpubl. manuscript), suggesting that most of the colonies used for pollination are likely to be infected by these pathogens. In fact, we observed the increase of *N. ceranae* loads in honey bees during strawberry pollination in greenhouses (Table 1). It demonstrates that *Nosema* microsporidia proliferates in the immune-suppressed honey bees that also undergo rapid aging by oxidative stress.

The mRNA levels for several cytochrome P450 genes (6AS10, 6AQ1, 6BD1, 9Q1, and 336A1) classified into the CYP3 clade are reduced in honey bees used for pollination in greenhouses (Fig. 6D). honey bees contain 46 cytochrome P450 genes, which are far fewer than the number in *Drosophila melanogaster* (85) and *Anopheles gambiae* (106) (Claudianos et al. 2006). Their physiological functions are not known; however, some members of the CYP3 clade are involved in pesticide detoxification in *A. gambiae* (Nikou et al. 2003), *Musca domestica* (Kasai and Scott 2001), and *Helicoverpa zea* (Sasabe et al. 2004). Intriguingly, these two honey bee cytochrome P450 genes, 6AQ1 and 6BD1, are orthologs of *D. melanogaster* CYP6G1 that is associated with dichloro-diphenyl-trichloro-ethane and neonicotinoid resistance (Daborn et al. 2002). Thus, downregulation of cytochrome P450 genes causes honey bees to become more susceptible to agrochemicals such as insecticides and fungicides, and may accelerate the collapse of colonies in greenhouses. In summary, the repression of immune system genes results in the accumulation of pathogens, and the downregulation of antioxidant system genes and genes associated with proteasome-dependent protein degradation accelerates honey bee senescence by accumulation of oxidized proteins. Furthermore, the suppression of cytochrome P450 family genes may render honey bees more susceptible to the toxicity of agrochemicals during long-term pollination in greenhouses.

What is the mechanism responsible for the downregulation of immune and antioxidant system genes, cytochrome P450 family genes, and genes associated with proteasome-dependent protein degradation in honey bees used for pollination? Because the functions of these genes are quite diverse, it is unlikely that a single pathway through the common *cis*-regulatory elements for mRNA transcription or degradation regulates their mRNA levels. Physiological changes initially induced by placing colonies in greenhouses may trigger alterations of multiple signaling pathways to reduce mRNA levels for the above genes. Interestingly, giving honey bees access to outside forage appears to reverse these effects. Nevertheless, it remains to be determined the identity of such physiological changes and signaling pathways.

## Figures and Tables

**Appendix A1:**
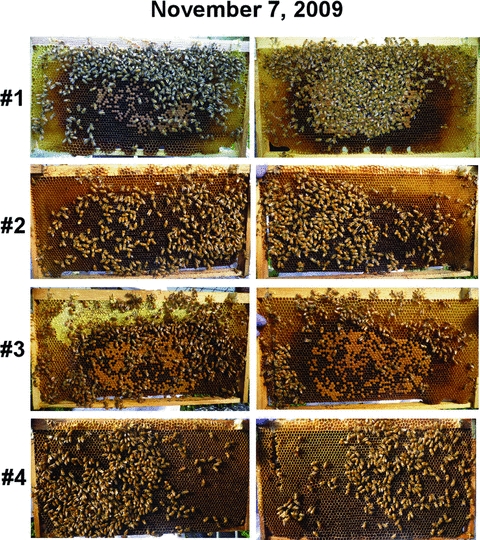
Honey bees in four-combs colony prior to installation in the strawberry greenhouse.

**Appendix A2:**
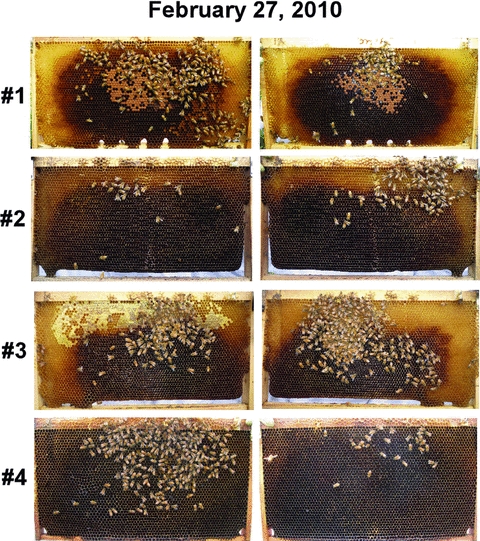
Honey bees in four-combs colony at 112 days after installation in the strawberry greenhouse.

**Appendix A3:**
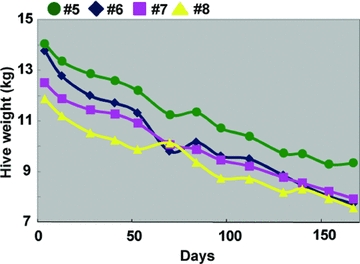
Changes in colony weight during pollination in strawberry greenhouses (November 5, 2010–April 21, 2011).

## Appendix

**Appendix A6:** Significantly enriched GO terms for honey bee genes differentially expressed during pollination in the strawberry greenhouses

GO ID	Level	GO Term	Count	%	*P*-value
GO:0007052	7,8,5,4,6	mitotic spindle organization	20	9.52	5.48E-06
GO:0016491	3	oxidoreductase activity	35	14.46	1.76E-05
GO:0007051	6,7,5	spindle organization	20	9.52	2.93E-05
GO:0000022	8,9,6,5,7	mitotic spindle elongation	12	5.71	3.10E-05
GO:0051231	7,8,5,4,6	spindle elongation	12	5.71	3.59E-05
GO:0016042	4,5	lipid catabolic process	7	3.33	9.38E-05
GO:0000226	5,4	microtubule cytoskeleton organization	22	10.48	0.000132
GO:0003746	5,4	translation elongation factor activity	5	2.07	0.0001903
GO:0006412	7,5,6	translation	27	12.86	0.0002655
GO:0000279	6,5	M phase	24	11.43	0.0003356
GO:0016616	5	oxidoreductase activity, acting on the CH-OH group of donors, NAD or NADP as acceptor	8	3.31	0.0006812
GO:0007010	4	cytoskeleton organization	28	13.33	0.0010085
GO:0022403	5,4	cell cycle phase	24	11.43	0.0010266
GO:0022402	4,3	cell cycle process	26	12.38	0.0010296
GO:0044242	5,6	cellular lipid catabolic process	5	2.38	0.0010766
GO:0016614	4	oxidoreductase activity, acting on CH-OH group of donors	9	3.72	0.001993
GO:0016115	7,8,5	terpenoid catabolic process	2	0.95	0.0020035
GO:0006719	8,7,10,9,6,5,11	juvenile hormone catabolic process	2	0.95	0.0020035
GO:0016803	5	ether hydrolase activity	2	0.83	0.0021799
GO:0007017	3	microtubule-based process	23	10.95	0.0022943
GO:0000302	5	response to reactive oxygen species	3	1.43	0.0026008
GO:0019843	5	rRNA binding	3	1.24	0.0029301
GO:0007049	3	cell cycle	28	13.33	0.003942
GO:0003824	2	catalytic activity	129	53.31	0.0042074
GO:0006732	5	coenzyme metabolic process	8	3.81	0.004423
GO:0004364	5	glutathione transferase activity	3	1.24	0.0044713
GO:0005198	2	structural molecule activity	21	8.68	0.0060481
GO:0016715	5	oxidoreductase activity, acting on paired donors, with incorporation or reduction of molecular oxygen, reduced ascorbate as one donor, and incorporation of one atom of oxygen	3	1.24	0.0063967
GO:0008135	4,3	translation factor activity, nucleic acid binding	8	3.31	0.0072627
GO:0050660	5,6	FAD binding	6	2.48	0.0076937
GO:0019538	5,4	protein metabolic process	63	30	0.0086252
GO:0004601	3,5	peroxidase activity	4	1.65	0.0089302
GO:0051187	5	cofactor catabolic process	4	1.9	0.0109526
GO:0006714	8,7,9,5	sesquiterpenoid metabolic process	2	0.95	0.0109757
GO:0006716	7,5,9,8,10,6	juvenile hormone metabolic process	2	0.95	0.0109757
GO:0015980	5	energy derivation by oxidation of organic compounds	8	3.81	0.015936
GO:0042743	5	hydrogen peroxide metabolic process	2	0.95	0.0174793
GO:0042744	5,6,8	hydrogen peroxide catabolic process	2	0.95	0.0174793
GO:0034599	5	cellular response to oxidative stress	2	0.95	0.0174793
GO:0016763	5	transferase activity, transferring pentosyl groups	3	1.24	0.0180175
GO:0016620	5	oxidoreductase activity, acting on the aldehyde or oxo group of donors, NAD or NADP as acceptor	3	1.24	0.0180175
GO:0044267	6,5	cellular protein metabolic process	50	23.81	0.0197558
GO:0008152	2	metabolic process	140	66.67	0.0225645
GO:0031032	6,5	actomyosin structure organization	4	1.9	0.0244822
GO:0008379	5,7	thioredoxin peroxidase activity	2	0.83	0.0270358
GO:0016755	5	transferase activity, transferring amino-acyl groups	2	0.83	0.0270358
GO:0044270	5	nitrogen compound catabolic process	4	1.9	0.0302641
GO:0042445	5,3	hormone metabolic process	3	1.43	0.0321093
GO:0044255	4,5	cellular lipid metabolic process	11	5.24	0.0321973
GO:0070011	5	peptidase activity, acting on L-amino acid peptides	16	6.61	0.033343
GO:0051339	5	regulation of lyase activity	2	0.95	0.0335132
GO:0004091	5	carboxylesterase activity	5	2.07	0.0345949
GO:0030163	6,5	protein catabolic process	18	8.57	0.0399476
GO:0007620	4,5	copulation	2	0.95	0.0426957
GO:0006890	7,5,6	retrograde vesicle-mediated transport, Golgi to ER	2	0.95	0.0426957
GO:0008061	5	chitin binding	4	1.65	0.0447769
GO:0008209	7,6,5	androgen metabolic process	1	0.48	0.0448622
GO:0008210	7,6,5	estrogen metabolic process	1	0.48	0.0448622
GO:0034723	6,7,5	DNA replication-dependent nucleosome organization	1	0.48	0.0448622
GO:0006844	5,6	acyl carnitine transport	1	0.48	0.0448622
GO:0015697	5,7,6,8	quaternary ammonium group transport	1	0.48	0.0448622
GO:0048791	6,7,8,9,5	calcium ion-dependent exocytosis of neurotransmitter	1	0.48	0.0448622
GO:0034308	5	monohydric alcohol metabolic process	1	0.48	0.0448622
GO:0046485	6,5,7	ether lipid metabolic process	1	0.48	0.0448622
GO:0006097	6,5,8	glyoxylate cycle	1	0.48	0.0448622
GO:0046333	6,8,9,7,5	octopamine metabolic process	1	0.48	0.0448622
GO:0015879	6,5,8,7,9	carnitine transport	1	0.48	0.0448622
GO:0006862	5,6	nucleotide transport	1	0.48	0.0448622
GO:0043059	5,4	regulation of forward locomotion	1	0.48	0.0448622
GO:0050661	5	NADP or NADPH binding	2	0.83	0.0458871
GO:0019211	4,5	phosphatase activator activity	1	0.41	0.0467814
GO:0016774	5	phosphotransferase activity, carboxyl group as acceptor	1	0.41	0.0467814
GO:0005172	5	vascular endothelial growth factor receptor binding	1	0.41	0.0467814
GO:0008160	5,6	protein tyrosine phosphatase activator activity	1	0.41	0.0467814
GO:0035035	5	histone acetyltransferase binding	1	0.41	0.0467814
GO:0008609	5	alkylglycerone-phosphate synthase activity	1	0.41	0.0467814
GO:0046923	5	ER retention sequence binding	1	0.41	0.0467814

**Appendix A7:** Significantly enriched GO terms for honey bee genes differentially expressed during pollination in the eggplant greenhouse

GO ID	Level	GO Term	Count	%	*P*-value
GO:0008236	6,5	serine-type peptidase activity	6	10.53	0.0004366
GO:0045087	4,5	innate immune response	3	6.12	0.002487
GO:0030163	6,5	protein catabolic process	8	16.33	0.0071401
GO:0016192	3,4,5	vesicle-mediated transport	8	16.33	0.0086862
GO:0070011	5	peptidase activity, acting on L-amino acid peptides	7	12.28	0.009631
GO:0007289	5,7,8,9,6	spermatid nucleus differentiation	1	2.04	0.0104678
GO:0009407	5,6	toxin catabolic process	1	2.04	0.0104678
GO:0042178	5,6	xenobiotic catabolic process	1	2.04	0.0104678
GO:0047396	4,5	glycosylphosphatidylinositol diacylglycerol-lyase activity	1	1.75	0.0110188
GO:0045793	5	positive regulation of cell size	2	4.08	0.0111832
GO:0043285	5	biopolymer catabolic process	8	16.33	0.0124452
GO:0002759	7,5,6,4	regulation of antimicrobial humoral response	2	4.08	0.0125466
GO:0002920	6,5	regulation of humoral immune response	2	4.08	0.0125466
GO:0002831	5,4	regulation of response to biotic stimulus	2	4.08	0.0125466
GO:0006325	5	establishment or maintenance of chromatin architecture	4	8.16	0.0148176
GO:0051187	5	cofactor catabolic process	2	4.08	0.018604
GO:0050776	5,4	regulation of immune response	2	4.08	0.0202579
GO:0035187	4,5,3	hatching behavior	1	2.04	0.020721
GO:0016030	5	metarhodopsin binding	1	1.75	0.0217989
GO:0005520	5	insulin-like growth factor binding	1	1.75	0.0217989
GO:0045927	5,3,4	positive regulation of growth	2	4.08	0.0237184
GO:0043900	4,3	regulation of multi-organism process	2	4.08	0.0237184
GO:0007043	5	cell-cell junction assembly	2	4.08	0.0255206
GO:0016042	4,5	lipid catabolic process	2	4.08	0.0273683
GO:0050778	6,4,5	positive regulation of immune response	1	2.04	0.0307626
GO:0002684	5,3,4	positive regulation of immune system process	1	2.04	0.0307626
GO:0035006	5,6	melanization defense response	1	2.04	0.0307626
GO:0002921	7,5,6	negative regulation of humoral immune response	1	2.04	0.0307626
GO:0008348	8,6,7,4,5	negative regulation of antimicrobial humoral response	1	2.04	0.0307626
GO:0002832	6,4,5	negative regulation of response to biotic stimulus	1	2.04	0.0307626
GO:0031349	6,7,4,5	positive regulation of defense response	1	2.04	0.0307626
GO:0031629	7,6,8,9,10,5	synaptic vesicle fusion to presynaptic membrane	1	2.04	0.0307626
GO:0045089	7,8,5,6	positive regulation of innate immune response	1	2.04	0.0307626
GO:0046844	6,7,4,8,9,5,10	micropyle formation	1	2.04	0.0307626
GO:0048489	4,5,6	synaptic vesicle transport	3	6.12	0.0323613
GO:0044270	5	nitrogen compound catabolic process	2	4.08	0.0331639
GO:0009404	4,5	toxin metabolic process	1	2.04	0.0405959
GO:0009253	5,6	peptidoglycan catabolic process	1	2.04	0.0405959
GO:0000270	5	peptidoglycan metabolic process	1	2.04	0.0405959
GO:0017143	5,6	insecticide metabolic process	1	2.04	0.0405959
GO:0006805	4,5	xenobiotic metabolic process	1	2.04	0.0405959
GO:0050962	8,6,5,7	detection of light stimulus involved in sensory perception	2	4.08	0.0414121
GO:0006897	5,4,6	endocytosis	5	10.2	0.0418106
GO:0016421	5	CoA carboxylase activity	1	1.75	0.0426585
GO:0015250	5,7	water channel activity	1	1.75	0.0426585
GO:0009583	5,6	detection of light stimulus	2	4.08	0.0435555
GO:0016811	5	hydrolase activity, acting on carbon-nitrogen (but not peptide) bonds, in linear amides	2	3.51	0.0451763
GO:0044255	4,5	cellular lipid metabolic process	4	8.16	0.0477807

## Supporting Information

**Table S4.** List of down- or upregulated honey bee genes during pollination in the strawberry greenhouses.

**Table S5.** List of down- or upregulated honey bee genes during pollination in the eggplant greenhouse.

**Table S8.** Significantly enriched GO terms for downregulated honey bee genes during pollination in the strawberry greenhouses.

**Table S9.** Significantly enriched GO terms for upregulated honey bee genes during pollination in the strawberry greenhouses.

**Table S10.** Significantly enriched GO terms for downregulated honey bee genes during pollination in the eggplant greenhouse.

**Table S11.** List of primers used for quantitative RT-PCR.
